# The prevalence of redundant nerve roots in patients with lumbar spinal stenosis is body position dependent: a retrospective observational study with repeated measures design in an upright MRI scanner

**DOI:** 10.1007/s00234-020-02423-x

**Published:** 2020-04-21

**Authors:** Luca Papavero, Stella Ebert, Carlos J. Marques

**Affiliations:** 1 Clinic for Spine Surgery, Schoen Clinic Hamburg Eilbek, Academic Hospital of the Medical Center Eppendorf (UKE), Hamburg, Germany; 2 Privatpraxis Upright-MRT, Hamburg, Germany; 3 Science Office of the Orthopedic and Joint Replacement Department, Schoen Clinic Hamburg Eilbek - Science Office, Dehnhaide 120, D-22081 Hamburg, Germany

**Keywords:** Redundant nerve roots, Central lumbar spinal stenosis, Dural cross-sectional area, Upright MRI

## Abstract

**Purpose:**

Redundant nerve roots (RNRs) are a negative prognostic factor in patients with central lumbar spinal stenosis (LSS). Forty percent of candidates for surgical decompression show RNRs (RNR+) on preoperative conventional magnetic resonance imaging (MRI). We investigated the prevalence of RNRs in three functional postures (standing, neutral sitting and flexed sitting) with an upright MRI (upMRI).

**Methods:**

A retrospective observational study with a repeated measures design. Thirty surgical candidates underwent upMRI. Sagittal and axial T2-weighted images of the three functional postures were evaluated. The segmental length of the lumbar spine (sLLS), the lordotic angle (LA) and the dural cross-sectional area (DCSA) were measured in each body position. Generalized linear mixed models were carried out. The 0.05 level of probability was set as the criterion for statistical significance.

**Results:**

The prevalence of RNRs decreased from 80% during standing to 16.7% during flexed sitting (*p* < 0.001). The sLLS increased significantly from standing to neutral sitting in both RNR groups (*p* < 0.001). The increase from neutral sitting to flexed sitting was only significant (*p* < 0.001) for the group without RNRs (RNR−). The LA decreased significantly for both RNR groups from standing to flexed sitting (*p* < 0.001). The DSCA increased significantly in the RNR− group (*p* < 0.001) but not in the RNR+ group (*p* = 0.9).

**Conclusion:**

The prevalence of RNRs is body position dependent. Increases in DCSA play a determinant role in resolving RNRs.

## Introduction

Patients with central lumbar spinal stenosis (LSS) scheduled for decompression surgery frequently show redundant nerve roots (RNRs) on preoperative magnetic resonance imaging (MRI). The prevalence rate of RNRs among patients with LSS ranges from 15 [1] to 45.3% [2], with most studies reporting a prevalence rate of approximately 40% [3–6].

**Fig. 1:**
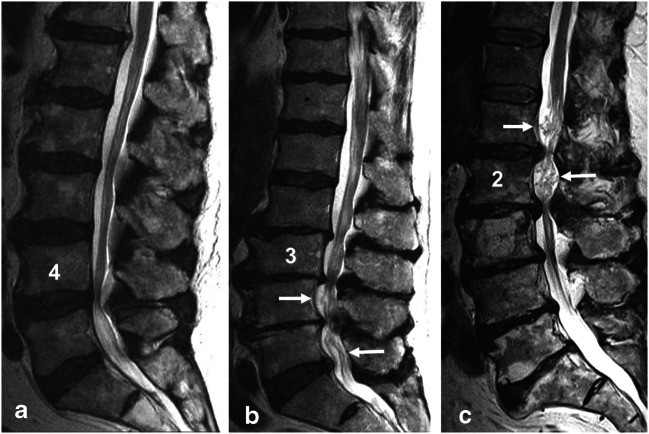
Sagittal T2-weighted images of the lumbar spine showing cauda equina nerve roots: **a** with no evidence of RNRs since the morphology of the cauda nerve roots is not affected by the stenosis at level L4/L5; **b** with serpentine-shaped RNRs (white arrows) caudally to the stenosis at level L3/L4 and **c** with loop-shaped RNRs (white arrows) cranially to the stenosis at level L2/L3

RNRs are described in the literature as tortuous, large, thickened and elongated nerve roots of the cauda equina, which can present in a serpentine or a loop shape [7] (Fig. 1). RNRs were observed cranially to the stenotic level in approximately 80% of the patients [1, 8], but they can also appear caudally to the narrowed segment or both [8, 9].

Patients with evidence of RNRs on preoperative MRI are older, have a smaller dural sac cross-sectional area (DCSA) at the stenotic level and have longer symptom duration. After decompression surgery, these patients had worse clinical scores and lower recovery rates than patients without RNRs [10]. Therefore, RNRs are considered a negative prognostic factor in patients with LSS.

Despite the clinical significance, the aetiology of RNRs is still unclear. In a historical case report, it was hypothesized that a compressive force squeezed the dural sac and caused the serpentine myelographic defects by displacing most of the RNRs in one direction [11]. Other authors hypothesized that multiple factors might contribute to the pathogenesis of RNRs, such as the extent of stenosis, age-dependent shortening of the spinal canal and dynamic or postural factors [2]. These authors concluded that RNRs are related to the spinal ageing process as well as to the mechanical friction at the stenotic level. However, the most accepted explanation model is the “squeeze theory” by Suzuki et al. [6]. The authors hypothesized that with age, the nerve roots are gradually squeezed out through the constriction. As an effect of the squeezing force, the nerve roots of the cauda become elongated and thickened.

In a study on potential predictors of RNRs in patients with LSS, several factors were identified as significant predictors [12]. The strongest predictors were LSS grade D and C according to Schizas [13], the number of stenotic levels involved and a decrease in the relative length of the lumbar spine. These results confirmed that multiple factors are involved in the aetiology of RNRs.

The primary aim of the study was to test whether body position is associated with the prevalence of RNRs in patients affected by LSS. We also investigated the effects of changes in body position (BP) on the segmental length of the lumbar spine (sLLS), dural cross-sectional area (DCSA) and lordotic angle (LA).

## Methods

### Study design

**Fig. 2:**
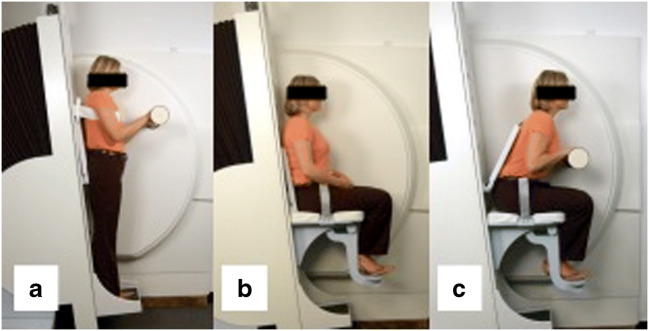
Upright MRI scanner enables the examination of the patient in different body positions. In the present study, patients were scanned in positions **a**, **b** and **c** during a single examination

This is a retrospective observational study with a repeated measures design. The database of a radiology centre was searched for upright MRI files of patients with LSS who were examined in three body positions during a single session: standing, neutral sitting and flexed sitting (Fig. 2). Sagittal and axial T2-weighted images were evaluated. The MRI images were anonymized for study purposes. Only limited data was available on patient demographics and clinical symptoms.

According to the Ethics Committee of the Federal State of Hamburg, Germany, retrospective observational studies do not require approval and patient informed consent whenever the data are acquired, saved and treated anonymously. This applies to the present study.

### Study sample

The sample consisted of 30 patients affected by LSS who were examined preoperatively with an upright MRI scanner (FONAR Upright™ MRI, 0.6 T; FONAR Corporation; Melville; NY 11747, USA). All patients were examined in a single private radiology office between 2015 and 2019. The most common reasons for the upright MRI examination were claustrophobia or obesity. The inclusion criteria were symptomatic lumbar central spinal stenosis with no previous lumbar spine surgery.

Patients were excluded if data in one or two body positions was not available.

A post hoc statistical power analysis was performed with the use of the software program G*Power (Psychology Department, Heinrich Heine University Düsseldorf, Germany) [14]. The primary question of the study was investigated with a chi-square test. The effect size was calculated based on the data presented in Table 2. Given an effect size of 1.604, an alpha error probability of 0.05 and a sample size of 30 patients, the achieved power for the analysis of the primary research question was 1.

### Assessment of the study variables

The sLLS was measured on T2-weighted sagittal MRI images. A line was drawn from the posterior-superior corner of the L1 vertebral body to the posterior-superior corner of the L2 vertebral body. The procedure was repeated until the line reached the posterior-superior corner of the S1 vertebral body. The length of the line was measured in millimetres (mm) [15]. A senior spine surgeon performed all measurements with the use of the AGFA Impax 6 software program (AGFA Health Care, GmbH, Bonn, Germany). The intra-rater reliability for sLLS measurements was tested previously. The estimated intraclass correlation coefficient (ICC) calculated with a two-way mixed effects model with an absolute agreement definition was 0.99 (95% CI ranging from 0.98 to 0.99).

The DCSA was measured on T2-weighted axial MRI images according to the description by Lim et al. [16]. The same neurosurgeon performed all measurements. The previously estimated intra-rater reliability was ICC = 0.96 (0.94 to 0.97).

The lumbar lordotic angle (LA) between the upper endplate of L1 and the lower endplate of L5 was measured on T2-weighted sagittal MRI images. The previously estimated intra-rater reliability was ICC = 0.97 (0.96 to 0.98).

Additionally, the MRIs were screened in each body position for the presence or absence of RNRs. The definition of RNRs presented with the ASED classification [17] for RNRs was used.

### Statistical analysis

A chi-square test was used to test whether the prevalence of RNR was associated with the repeated measures factor “body position”. Generalized linear mixed models (GLMMs), with data ordered in a “stacked” form, were used to test for significant effects of the between-group factor “RNR” (RNR+ vs. RNR−) and the within-group factor “body position” (repeated measures factor) on the dependent variables, sLLS, LA and DCSA. Multiple comparisons between the pairs of estimated means were conducted with paired *t* tests using the LSD adjustment of α for the within-subject factor “body position”. In case a significant main effect for the between-subject factor was found, comparisons between the groups were carried out with a *t* test for independent samples in each body position.

Pearson’s correlation was used to test the relationship between the LA and DCSA, age and neurogenic claudication and DCSA and neurogenic claudication.

The IBM SPSS software version 21 for Macintosh (IBM Corp. Armonk, New York) was used for all statistical analyses. For all statistical tests, the 0.05 level of probability was set as the criterion for statistical significance.

## Results

The mean age of the patients in the sample was 68.2 ± 8.0 years. Fourteen patients (43.8%) had back pain symptoms, of which 10 patients (33.3%) had additional leg pain symptoms (Table 1).

**Table 1:** Symptoms of the patients before MRI examination

Symptoms	yes	no
Back pain	14 (43.8)	14 (43.8)
Leg pain	17 (53.1)	11 (34.4)
Neurogenic claudication	17 (53.1)	11 (34.4)

Values are frequencies and (%). For two patients in the sample, symptom data were not available

**Fig. 3:**
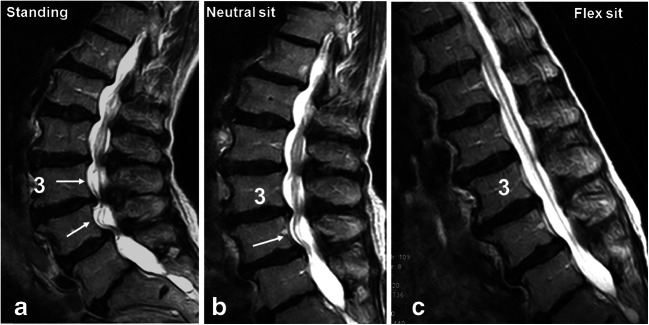
Sagittal T2-weighted upright MRI images of a single patient: **a** in standing position with evidence of serpentine-shaped RNRs (white arrows) cranially and caudally from the stenotic level L3/L4; **b** in neutral sitting position with RNRs (white arrow) only caudally from the stenotic level and **c** in flexed sitting position with no evidence of RNRs

There was a statistically significant association between body position and the prevalence of RNRs on the MRI images (*x*^2^ = 28.3, *p* < 0.001) (Table 2). The prevalence of RNRs decreased by 66.6% with the change from standing to neutral sitting and decreased further by 37.5% with the change from neutral sitting to flexed sitting. Accordingly, the number of patients with no evidence of RNR on their MRI images (RNR−) increased with the change from the standing position through both sitting positions (Fig. 3 a–c).

**Table 2:** Prevalence of RNR in 30 patients across body positions

	Standing	Neutral sitting	Flexed sitting
RNR+	24 (80.0)	8 (26.7)	5 (16.7)
RNR−	6 (20.0)	22 (73.3)	25 (83.3)

Values are frequencies and (%) for patients with (+) and without (−) evidence of redundant nerve roots (RNR)

The estimated means with 95% CI for the dependent variables sLLS, DCSA and LA are presented in Table 3.

**Table 3:** Descriptives

		sLLS (mm)	DCSA (mm^2^)	Lordotic angle (degrees)
Standing	RNR+	159.5 [134.9–184.1]	68.8 [− 7.5–145.2]	49.8 [24.3–75.3]
	RNR−	159.2 [134.5–183.9]	59.9 [− 18.3–138.3]	47.0 [20.9–73.1]
Neutral sitting	RNR+	165.8 [141.1–190.5]	79.8 [2.0–157.5]	26.9 [1.0–52.9]
	RNR−	164.2 [139.5–188.8]	83.9 [7.5–160.4]	28.7 [3.2–54.3]
Flexed sitting	RNR+	167.6 [142.8–192.3]	81.3 [2.3–160.3]	14.5 [− 11.8–40.8]
	RNR−	168.6 [144.0–193.2]	107.8 [31.4–184.1]	13.2 [− 12.2–38.7]

Values are mean and [95% CI of the mean] for segmental length of lumbar spine (sLLS), dural cross-sectional area (DCSA) of the key stenotic level and lordotic angle (LA)

For the variable sLLS, the model was significant (*F*_(5)_ = 35.9, *p* < 0.001), and there was a significant main effect for body position (*F*_(2)_ = 37.7, *p* < 0.001) and no significant effect for the RNR group (*F*_(1)_ = 0.08, *p* = 0.7). The sLLS increased significantly from standing to neutral sitting in both RNR groups (*p* < 0.001). The increase in sLLS between neutral sitting and flexed sitting was significant for the RNR− group (*p* < 0.001) and non-significant for the RNR+ group (*p* = 0.2).

For LA, the model was also significant (*F*_(5)_ = 74.9, *p* < 0.001). There was a significant main effect for “body position” (*F*_(2)_ = 80.8, *p* < 0.001) and no significant effect for the RNR group (*F*_(1)_ = 0.07, *p* = 0.7). In both RNR groups, the LA decreased significantly from standing to neutral sitting (*p* < 0.001) and from neutral sitting to flexed sitting (*p* = 0.006 and *p* < 0.001 for RNR+ and RNR−, respectively).

**Fig. 4:**
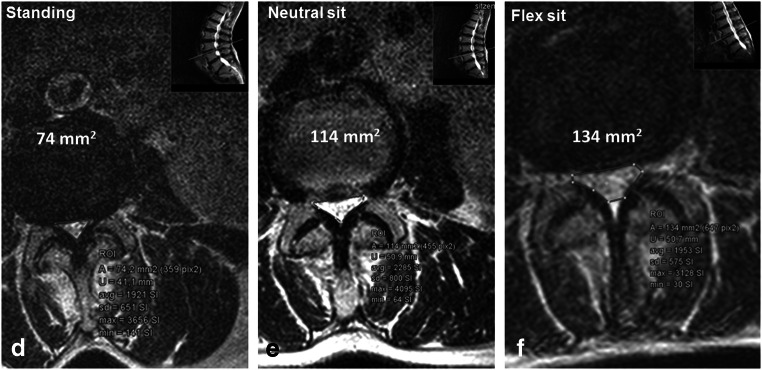
Axial T2-weighted images of the same patient at the stenotic level L3/L4 presenting the measurement of the DSCA in **d** standing, **e** neutral sitting and **f** flexed sitting positions

**Fig. 5:**
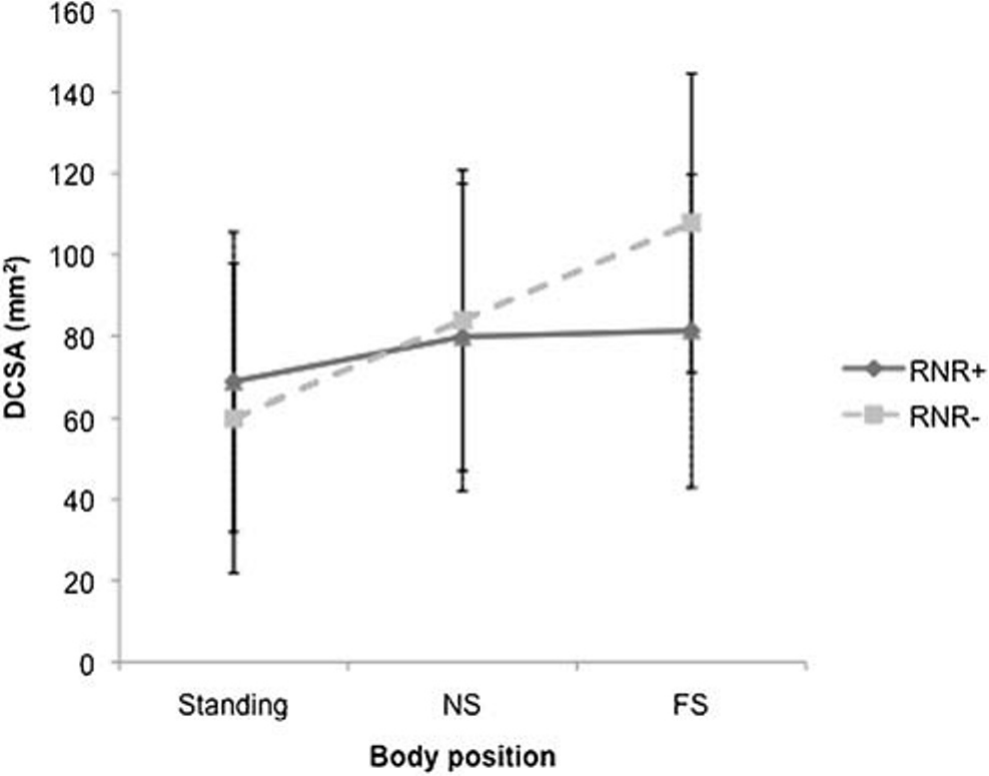
Estimated mean DCSA (mm^2^) with standard error of the mean (SEM) for RNR groups across body positions (NS, neutral sitting; FS, flexed sitting)

For DCSA, the model was also significant (*F*_(5)_ = 8.9, *p* < 0.001). The effects were significant for body position (*F*_(2)_ = 6.4, *p* = 0.003) and not significant for the RNR group (*F*_(1)_ = 0.76, *p* = 0.3). The DCSA increased significantly by 23.9 mm^2^ from standing to neutral sitting (*p* = 0.04) and by 23.8 mm^2^ from neutral sitting to flexed sitting (*p* = 0.001) in the RNR− group (Fig. 4 d–f). For the RNR+ group, the DCSA increased by 10.9 mm^2^ from standing to neutral sitting (*p* = 0.2) and by 12.5 mm^2^ from neutral sitting to flexed sitting (*p* = 0.9), but these differences were not statistically significant (Fig. 5).

A weak negative relationship was found between LA and DCSA (*r* = − 0.23, *p* = 0.04). There was neither a significant correlation between patients’ age and neurogenic claudication symptoms (*r* = 0.06, *p* = 0.7) nor between DCSA and neurogenic claudication symptoms (*r* = − 0.03, *p* = 0.8).

## Discussion

In patients affected by central LSS, the symptoms frequently worsen with lumbar extension (standing or walking), whereas they resolve with lumbar flexion (stooping or sitting). In the early 1980s, when myelography was the gold standard for imaging LSS, flexion-extension myelograms showed that flexion improved the passage of contrast medium. Patients with a complete block were left in the flexed sitting position until the contrast medium passed the block and showed the caudal portion of the dural sac [18]. Suzuki et al. observed that in functional myelograms, the number of RNRs decreased slightly during flexion but increased substantially during extension [6]. This observation led to the pathogenetic “squeeze theory” of RNRs, in which it was assumed that repeated flexion-extension of the lumbar spine squeezes the cauda nerve roots gradually through the constriction of the stenotic level and that these forced out roots become thickened and elongated [6]. This pathogenetic mechanism of “friction neuritis” is still widely accepted. In a previous investigation, Lao et al. [19] demonstrated a significant correlation between a decrease of more than 30% in the DCSA, measured in “standing” MRI images, and an extremely shortened walking distance in patients with LSS.

Clinical (longer history, more severe claudication and less benefit from surgical decompression), electrophysiological (lower nerve conduction velocity, irregular action potentials and nocturnal leg cramps) and histopathological (demyelination and axonal loss) factors categorize RNRs as a negative prognostic factor [6]. The present upright MRI study focused on the dynamic changes of the RNRs. A functional posture that aggravates LSS-related symptoms (standing) was compared with two functional postures (neutral and flexed sitting) that cause relief of symptoms. The results showed a strong association between DCSA and the prevalence of RNRs. The prevalence of RNRs dropped from 80% during standing to 16.7% during flexed sitting. The role of DCSA as the strongest predictor of RNRs [12] was confirmed.

**Fig. 6:**
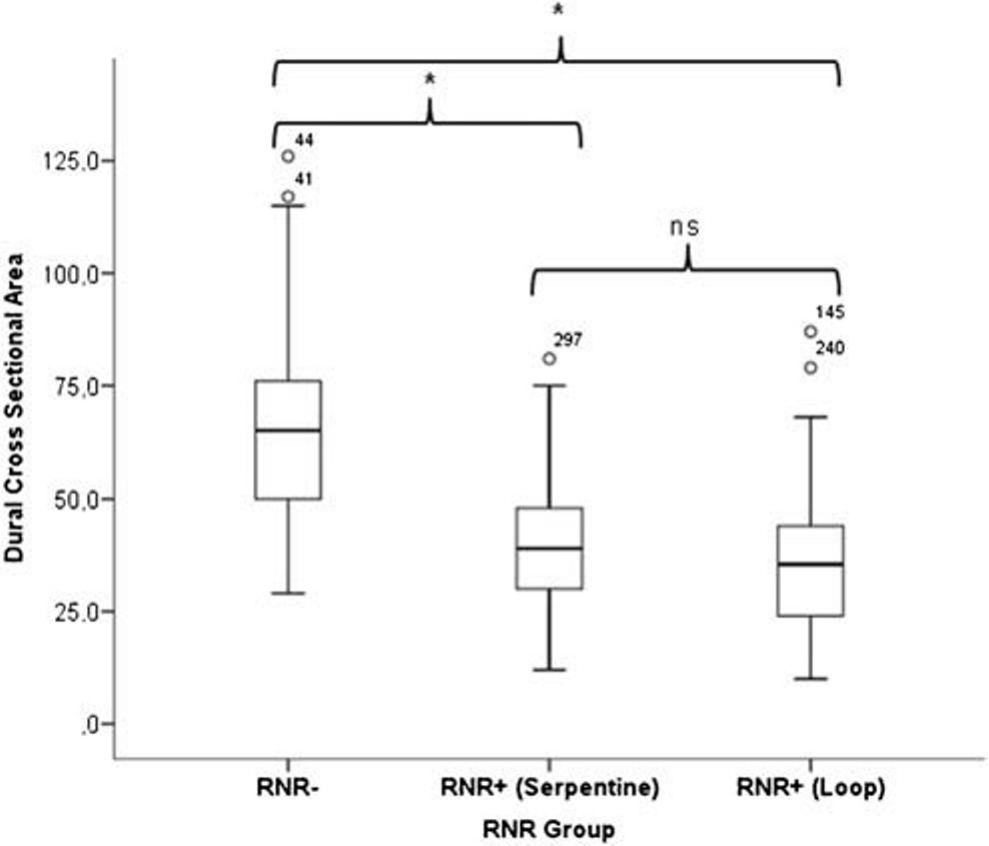
Dural cross-sectional area (DCSA) (mm^2^) of 141 LLS patients without evidence of RNR (RNR−), 58 patients with evidence of serpentine-shaped RNR (RNR+ serpentine) and 110 patients with evidence of loop-shaped RNR (RNR+ loop) (*significant difference for *p* < 0.001; ns, non-significant)

The causal relationship between the DCSA of the key stenotic level and the prevalence of RNRs was also confirmed by measurements that we performed in patients who were examined in a lying position in a conventional closed bore 1.5-T MRI scanner. The mean DCSA of 141 patients without evidence of RNR (RNR−) (65 ± 19 mm^2^) was significantly (*p* < 0.001) greater than the mean DCSA of 58 patients with evidence of serpentine-shaped RNR (RNR+) (40 ± 16 mm^2^) and 110 patients with evidence of loop-shaped RNR (RNR+) (36 ± 15 mm^2^). The mean difference in DSCA between the two RNR+ subgroups was not statistically significant (*p* = 0.2). The mean DCSA of the whole group of RNR+ patients was 38 ± 18 mm^2^ (Fig. 6). These figures derive from unpublished data, but since they reinforce the findings of the present study, we have used them to discuss our findings.

Furthermore, the prevalence of RNRs also decreased due to the simultaneous increase of the sLLS and the decrease of the LA with the change from standing to neutral sitting to flexed sitting. The treatment effect of interspinous devices can be explained by this mechanism.

The three key findings of this study are as follows: first, RNRs are a functional feature of lumbar spine stenosis and are body position dependent; second, the DCSA is the most important factor regulating the prevalence of RNRs and third, RNRs are diseased cauda nerve roots that strongly affect patients with LSS. The antalgic trunk flexion during walking, the relief provided by sitting or stooping and the progressive shortening of the walking distance are self-help mechanisms that decrease the prevalence of RNRs.

The image acquisition time of the three-body positions was approximately 45 min. There were no MRI files in the searched database containing an additional scan in the supine position. A post hoc database search was performed aiming to gather MRI files of patients with LLS and evidence of RNR who were scanned in standing, supine and neutral sitting positions in a single session. One hundred forty-two patient files of those examined in these three positions in a single session were found. Of these, eight patients had LLS and presented evidence of RNRs on their MRI images. In this small group of patients, the prevalence of RNRs was also body position dependent (*x*^2^ = 16.4, *p* < 0.001). There was evidence of RNRs in the MRI images of all eight patients (100%) in standing position. The prevalence of RNR+ was 37.5% in the supine and 0% in neutral sitting positions (Table 4). Unfortunately, there were no axial images available in the supine position. Hence, the DCSA in the supine position could not be measured. Due to the reduced number of cases, GLMM could not be applied and no further data analysis was performed.

**Table 4:** Prevalence of RNR in 8 patients across three body positions

	Standing	Supine	Neutral sitting
RNR+	8 (100)	3 (37.5)	0 (0)
RNR−	0 (0)	5 (62.5)	8 (100)

Values are frequencies and (%) for patients with (+) and without (−) evidence of redundant nerve roots (RNR)

If available, it would have been interesting to evaluate the scans of patients in all four body positions obtained in a single session. This can be seen as a study limitation.

This is a observational retrospective study. The database of a private radiology office was searched for image files. To comply with the ethical standards, all datasets were anonymized before they were sent to us. Therefore, only restricted preoperative and no postoperative clinical data of the patients were available. This study limitation was acceptable as the investigation of the relationships between imaging and clinical data was not the aim of this work.

## Conclusions

The frequency and shape of RNRs depend on body position. DCSA affects the prevalence of RNRs. The spontaneous antalgic behaviour of patients affected by LSS leads to a DCSA increase and reduces the prevalence of RNRs.
